# Long Noncoding RNA LINC01554 Inhibits the Progression of NSCLC Progression by Functioning as a ceRNA for miR-1267 and Regulating ING3/Akt/mTOR Pathway

**DOI:** 10.1155/2022/7162623

**Published:** 2022-07-08

**Authors:** Zizong Wang, Bin Yang, Jin Zhang, Xiangyang Chu

**Affiliations:** ^1^Department of Thoracic Surgery, The First Medical Center, Chinese PLA General Hospital, Beijing 100853, China; ^2^Qingdao University, Qingdao, Shandong 266071, China; ^3^Department of Integrated Oncology Treatment, Qingdao University Affiliated Hospital, Qingdao, Shandong 266000, China

## Abstract

**Objectives:**

This study focused on the biological functions and mechanisms of action of LINC01554 in nonsmall cell lung cancer (NSCLC).

**Methods:**

The expression and prognostic values of LINC01554 in NSCLC were evaluated using The Cancer Genome Atlas datasets. MTT, colony formation, wound healing, transwell, and in vivo assays were performed to investigate the role of LINC01554 in NSCLC. The related protein expression levels were measured via western blotting. Bioinformatic analysis was conducted to predict targeted genes. The relationship between LINC01554, microRNA- (miR-) 1267, miR-1267, and inhibitor of growth family member 3 (ING3) was analysed via a dual-luciferase reporter assay.

**Results:**

LINC01554 expression was downregulated in NSCLC and associated with NSCLC prognosis. LINC01554 overexpression suppressed NSCLC cell proliferation, migration, invasion, and epithelial-mesenchymal transition (EMT). Bioinformatic and dual-luciferase reporter assays demonstrated that LINC01554 expression directly targeted miR-1267 expression, which in turn directly acted on ING3. An miR-1267 mimic significantly reduced ING3 expression, whereas an miR-1267 inhibitor observably elevated its expression. LINC01554 overexpression increased ING3 expression, whereas this effect was counteracted by the miR-1267 mimic. LINC01554 overexpression also significantly suppressed the expression of phosphorylated protein kinase B (Akt) and phosphorylated mammalian target of rapamycin (mTOR) expression; this effect was abrogated by the miR-1267 mimic. Mechanistically, LINC01554 overexpression repressed the growth, migration, invasion, and epithelial-mesenchymal transition (EMT) of NSCLC cells through the regulation of the miR-1267/ING3 axis via regulation of the Akt/mTOR signalling pathway.

**Conclusions:**

We provide the first evidence of the involvement of the LINC01554/miR-1267 axis in NSCLC proliferation and metastasis through the ING3Akt/mTOR pathway. Thus, LINC01554 may serve as a novel therapeutic target for NSCLC.

## 1. Introduction

Lung cancer is the most common malignancy, accounting for approximately 11.6% of all malignant tumours worldwide [1, 2]. Nonsmall cell lung cancer (NSCLC) accounts for approximately 80–85% of all lung cancers. The main subtypes of NSCLC are lung adenocarcinoma (LUAD) and lung squamous cell carcinoma (LUSC) [3]. Despite considerable advances in diagnostic techniques and treatment strategies, the prognosis of NSCLC patients remains poor, with the 5-year overall survival rate being as low as 15% [4, 5]. Thus, there is an urgent need to understand the detailed molecular mechanism associated with NSCLC proliferation and metastasis and explore novel therapeutic targets for metastatic NSCLC treatment.

Recent evidence has highlighted the key role of ncRNAs (noncoding RNA) in tumorigenesis [6]. Two typical ncRNA subtypes are miRNAs (microRNAs) and long chain noncoding RNAs (lncRNAs) [7, 8]. It is believed that the interaction between lncRNAs and miRNAs may play an important role in tumorigenesis. Based on lncRNA-miRNA-mRNA network, important regulatory pathways and therapeutic targets can be revealed. For instance, the lncRNA JPX is associated with poor prognosis and progression of NSCLC through direct interaction with microRNA- (miRNA, miR-) 145-5p and *CCND2* [9]. The lncRNA MIR17HG inhibits proliferation, migration, and invasion of NSCLC cells by adsorbing miR-142-3p via sponging, thereby upregulating its expression and simultaneously downregulating *Bach-1* expression [10]. In addition, the expression of the lncRNA SBF2-AS1 is markedly elevated in NSCLC tissues, and it regulates NSCLC cell migration and invasion by modulating the miR-362-3p/growth factor receptor-bound protein 2 axis [11]. The expression of long intergenic nonprotein coding RNA 1554 (LINC01554), located on chromosome 5q15, was found to be downregulated in hepatocellular carcinoma (HCC) where it was involved in cancer progression [2, 12, 13]. However, the expression and biological functions of LINC01554 in NSCLC remain unclear.

The bioinformatics analysis shows that predicted that miR-1267 and inhibitor of growth family member 3 (ING3) were the downstream target of LINC01554. Further gene set enrichment analysis (GSEA) analysis found that LINC01554 inhibited the Akt/mTOR pathway based on the TCGA databases. Thus, in the current study, we explored whether LINC01554 could regulate NSCLC cell proliferation, migration, invasion, and EMT via functioning as a (competitive endogenous RNA) ceRNA for miR-1267 and by regulating the ING3/Akt/mTOR pathway.

## 2. Materials and Methods

### 2.1. Bioinformatic Analysis Based on the Cancer Genome Atlas (TCGA) Database

The lncRNAs, miRNAs, gene expression profiles, and clinical data of LUAD as well as LUSC were downloaded from TCGA-LUAD and TCGA-LUSC, respectively (http://portal.gdc.cancer.gov/). Differentially expressed lncRNAs, miRNAs, and mRNAs were obtained using the UALCAN (http://ualcan.path.uab.edu/) webtool. TCGA-LUSC dataset contained 551 tissue samples: 502 LUSD and 49 normal control tissues. TCGA-LUAD dataset included a total of 592 tissue samples (533 LUAD and 59 normal control tissues). The expression and prognostic values of LINC01554 in LUAD and LUSC patients were analysed using Kmplot (https://kmplot.com/analysis/). Based on TCGA-LUSC and TCGA-LUAD datasets, we also performed a GSEA using Guilt By Association Analysis (GTBAdb, http://guotosky.vip:13838/GTBA/).

### 2.2. Cell Culture and Transfection

NSCLC cell lines H23, H1299, NCI-H520, and A549 and the normal human bronchial epithelial cell line, 16HBE, were supplied by the American Type Culture Collection (Rockville, MD, USA) and cultured in Roswell Park Memorial Institute- (RPMI-) 1640 medium (Gibco, USA) supplemented with 10% foetal bovine serum (HyClone, Canada) at 37°C in 5% CO_2_ [14].

Vectors such as pcDNA3.1-LINC01554, the empty pcDNA3.1 vector, si-negative control (NC), si-LINC01554-1, and si-LINC01554-2 were synthesised by GenePharma (Shanghai, China) along with the miR-1267 mimic/inhibitor and NC mimic/inhibitor. Transfections were conducted using Lipofectamine 3000 (Invitrogen, CA, USA) following standard protocols. The culture medium was replaced with complete RPMI-1640 medium 6 h after transfection. Cells were collected after 48 h of transfection, and quantitative reverse transcription–polymerase chain reaction (RT-qPCR) was performed to detect LINC01554 and miR-1267 expression for the analysis of transfection efficiency.

### 2.3. RT-qPCR

Total RNA from cells was extracted using the TRIzol kit (Invitrogen, Carlsbad, CA, USA). A reverse transcription kit (Takara, Dalian, China) was used to synthesise the cDNA. Real-time PCR analyses were performed according to the instructions of the SYBR Premix Ex Taq kit (Takara, Otsu, Japan). The amplification conditions were as follows: 95°C for 10 min, 40 cycles of 95°C for 15 s, 61°C for 30 s, and 72°C for 30 s. The relative quantification of genes was assessed using the 2^−∆∆Ct^ method [15]. The primers used were as follows: LINC01554, GAGAGAGCCAACAGTCCAGG (forward) and GCTACTCTGGCACTCTGCAT (reverse); *ING3*, AGACACTCCTTCACAGCCAGT (forward) and CTTCGTCCCTCCTTCATCTGAG (reverse); miR-1267, ATCCAGTGCAGGGTCCGAGG (forward) and GCGGCGGCTCCCAAATCTCCTG (reverse); *GAPDH*, CATGTTGCAACCGGGAAGGA (forward) and GCCCAATACGACCAAATCAGAG (reverse).

### 2.4. Cell Proliferation Assay

Cell proliferation was assessed using the 3-(4,5-dimethylthiazol-2-yl)-2,5-diphenyl tetrazolium bromide (MTT) assay, as previously described [16]. Cells were plated in 96-well plates at a density of 1 × 10^3^ cells/well. Next, 20 *μ*L of MTT solution (Beyotime, Shanghai, China) was added to each well for 4 h. Absorbance was determined at 450 nm using a microplate reader (Molecular Devices, CA, USA).

### 2.5. Colony Formation Assay

As previously described, cells were seeded into six-well plates (1 × 10^2^ cells/well) and cultured [17]. Colonies with more than 50 cells were fixed using 4% formaldehyde for 15 min at 37°C. The colonies were subsequently stained using crystal violet (Beyotime, Shanghai, China). After 15 min, colonies were counted and photographed under a microscope (Bethesda, MD, USA).

### 2.6. Wound Healing Assay

Cells were plated into six-well plates containing serum-free medium until they reached at least 80% confluency. The cell layer was then scratched using a sterile 200 *μ*L pipette tip. Cells were washed with phosphate-buffered saline to remove any debris, and the cell culture medium was replaced. After 48 h of culture, representative images were obtained using an inverted light microscope (Olympus Corporation) [18].

### 2.7. Transwell Invasion Assay

Transwell chambers (Millipore, Billerica, MA, USA) containing Matrigel were used to determine the invasive ability of cells [16]. A total of 1 × 10^5^ cells in RPMI-1640 serum-free growth medium (Gibco, USA) were seeded in the upper wells, whereas the lower wells were filled with the same medium containing 10% serum. After 24 h incubation at 37°C, the cells invading the lower side of the chamber were fixed with 4% paraformaldehyde for 20 min and stained with 0.1% crystal violet for 10 min. Images were captured with a microscope (Olympus Corporation).

### 2.8. Western Blot Analysis

Total protein was extracted and separated using sodium dodecyl sulphate polyacrylamide gel electrophoresis (10–12% gels), and the separated protein bands were transferred onto polyvinylidene difluoride membranes (Millipore, USA). The membranes were blocked with 5% nonfat milk diluted in Tris-buffered saline with 0.05% Tween-20 and incubated with primary antibodies (E-cadherin, 1 : 1000, abs130068; snail, 1 : 1000, abs151371; vimentin, 1 : 1000, abs131996; glyceraldehyde 3-phosphate dehydrogenase (GAPDH), 1 : 1000, abs132004; ING3, 1 : 1000, abs137556; Akt, 1 : 1000, abs131788; p-Akt, 1 : 1000, abs130889; mTOR, 1 : 1000, abs131824; p-mTOR, 1 : 1000, abs130933) overnight at 4°C. After rinsing, the membranes were probed with a goat antirabbit antibody (1 : 2000, abs20040, Absin Bioscience, Shanghai, China) for 2 h. Protein signals were visualised by chemiluminescence using an enhanced chemiluminescence kit (Millipore, USA) [17].

### 2.9. Dual-Luciferase Reporter Assay

StarBase (http://starbase.sysu.edu.cn/) was used to predict the potential miRNAs that interact with LINC01554. TargetScan (http://www.targetscan.org/vert_71/) and StarBase tools were used to identify potential miR-1267 mRNA targets. The wild-type- (WT-) LINC01554, mutant-type- (MUT-) LINC01554, WT-ING3, and MUT-ING3 containing binding sites for miR-1267 were inserted into the pGL3 luciferase reporter vector (Promega, Madison, WI, USA). Subsequently, the reporter vector and miR-1267 mimic or negative control were transfected into H23 and H1299 cells using Lipofectamine 3000. After 48 h from transfection, the luciferase activity was assessed using a dual-luciferase detection kit (Promega Corporation) [16].

### 2.10. Lung Cancer Xenograft

Male BALB/c nude mice purchased from Jinan Pengyue Laboratory Animal Co., Ltd (Jinan, China) were divided into two groups (*n* = 4 per group) as follows: pcDNA3.1 LINC01554 and pcDNA3.1 NC. The animal experiments in this study were approved by the Animal Care and Use Committee of Chinese PLA General Hospital (S2021-001-12). To establish an in vivo tumour model, 5 × 10^6^ H23 cells pretransfected with pcDNA3.1 LINC01554 or pcDNA3.1 NC were subcutaneously injected into nude mice. Ten days after injection, tumour growth was recorded every 5 days. The length and width of tumours were calculated using callipers. Tumour volume was calculated as follows: tumour volume = (length × width^2^)/2. Mice were euthanised with prior anaesthesia by an intraperitoneal injection of 3% sodium pentobarbital (30 mg/kg) 30 days after injection [17].

### 2.11. Statistical Analyses

All experiments were repeated at least thrice. Data are shown as the mean ± standard deviation. Data were assessed by the Student's *t*-test when comparing two groups and one-way analysis of variance for experiments with more than two groups. Statistical significance was set at *P* < 0.05.

## 3. Results

### 3.1. LINC01554 Expression and Prognosis of NSCLC

**Figure 1:**
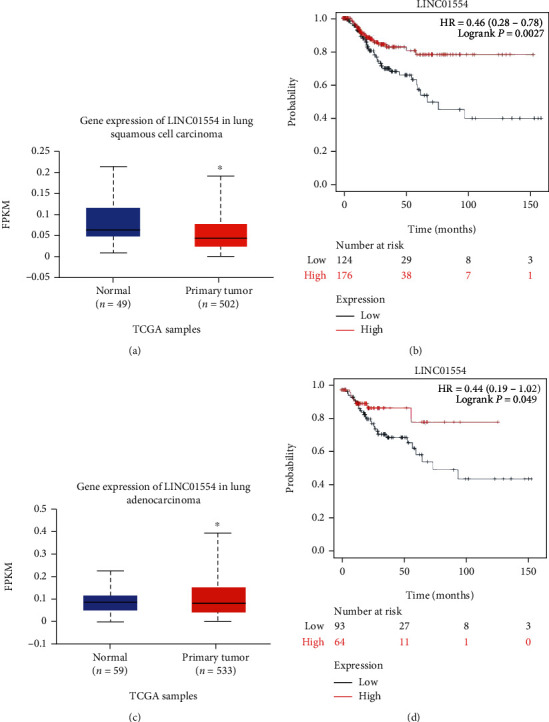
LINC01554 expression was downregulated in NSCLC and associated with its prognosis. (a) LINC01554 expression level in LUSC from TCGA database. (b) Survival analysis based on LINC01554 expression levels in LUSC patients from high and low expression groups. (c) LINC01554 expression level in LUAD from TCGA database. (d) Survival analysis based on LINC01554 expression levels in LUAD patients from high and low expression groups.

The comparative analysis of LUSC and LUAD tumour tissues and paracancerous tissues from TCGA database showed that the expression of LINC01554 was down regulated in tumours compared with normal tissues (Figures 1(a) and 1(c), *P* < 0.05). Based on the median expression of LINC01554, LUSC and LUAD specimens were divided into high and low LINC01554 groups. The survival analysis indicated that patients with lower expression of LINC01554 in LUSC and LUAD had a poorer prognosis and lower survival than those with higher LINC01554 expression (Figures 1(b) and 1(d), *P* < 0.05).

### 3.2. LINC01554 Overexpression Suppressed NSCLC Cell Proliferation In Vitro

**Figure 2:**
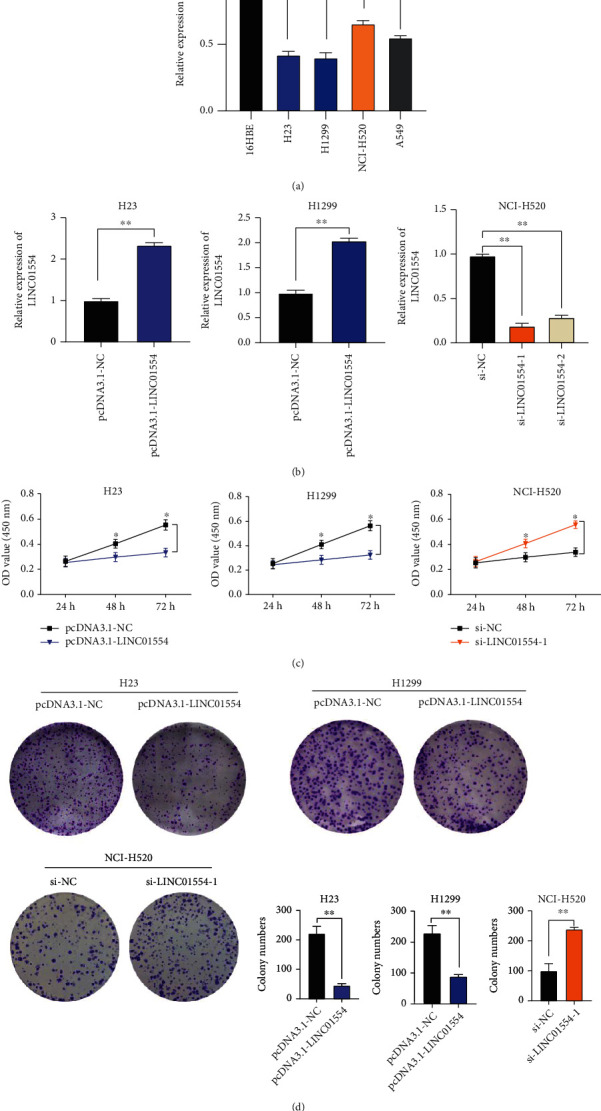
LINC01554 overexpression suppressed NSCLC cell proliferation. (a) The relative expression of LINC01554 in NSCLC cell lines and normal human bronchial epithelial cell lines was verified via RT-qPCR. (b) pcDNA3.1-LINC01554, the empty pcDNA3.1 vector, si-NC, and si-LINC01554 were transfected in H23, H1299, and NCI-H520 cells, and the transfection efficiency was determined via RT-qPCR. (c) Cell viability was tested via the MTT assay. (d) Cell vitality was detected via a colony formation assay. ∗*P* < 0.05, and ∗∗*P* < 0.01.

We tested LINC01554 expression level in normal 16HBE cells and NSCLC cells and found it to be significantly downregulated in H23 and H1299 cells as compared with that in 16HBE cells (Figure 2(a), *P* < 0.05). As shown in Figure 2(b), LINC01554 expression was notably elevated and suppressed following transfection with LINC01554-overexpressing vectors and small-interfering RNAs (siRNAs; si-LINC01554-1 and si-LINC01554-2), respectively (*P* < 0.05). The results of MTT and colony formation assays revealed that cell viability and proliferation were suppressed in LINC01554 overexpression group but elevated in the si-LINC01554 transfection group (Figures 2(c) and 2(d), *P* < 0.05).

### 3.3. LINC01554 Overexpression Suppressed NSCLC Cell Migration, Invasion, and EMT In Vitro

**Figure 3:**
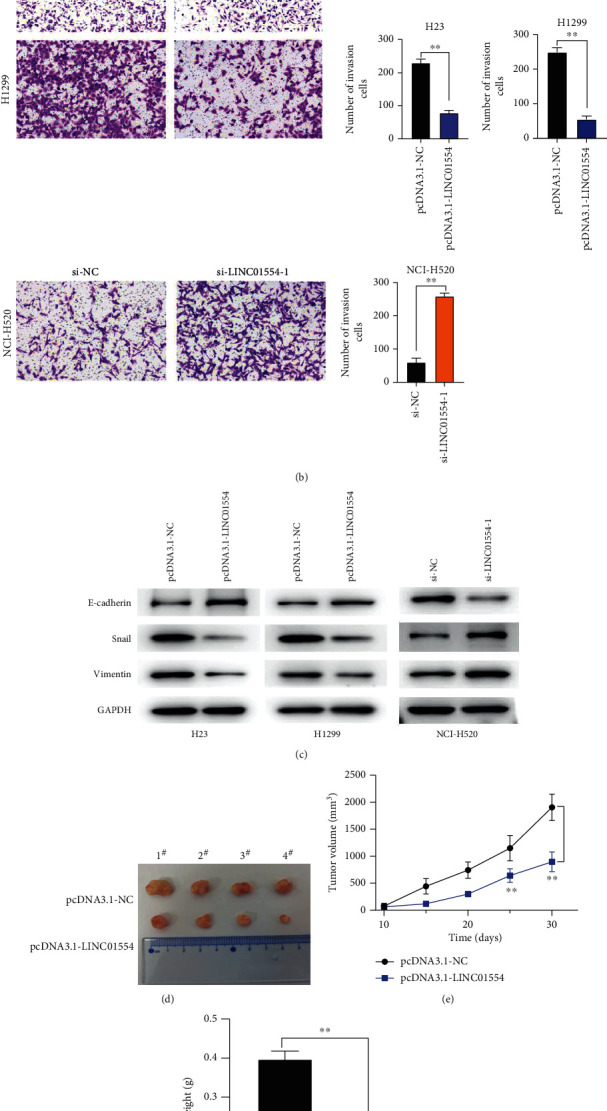
LINC01554 overexpression suppressed NSCLC cell migration and invasion. (a) Cell migration was detected and quantitated via the wound healing assay in H23, H1299, and NCI-H520 cells. (b) Cell invasion was tested and quantitated via the transwell assay in H23, H1299, and NCI-H520 cells. (c) E-cadherin, snail, and vimentin expression were detected and quantitated via western blotting. (d) The photograph of the tumours. (e) Tumour volume and (f) tumour weight were determined. ∗*P* < 0.05, and ∗∗*P* < 0.01.

As shown in Figures 3(a) and 3(b), the cell invasion capacity was significantly inhibited by LINC01554 overexpression (*P* < 0.05). Western blot analysis results of EMT-related protein expression in H23 and H1299 cells showed that the expression of E-cadherin evidently increased but that of snail and vimentin was markedly downregulated upon LINC01554 overexpression (Figure 3(c), *P* < 0.05). Nevertheless, LINC01554 knockdown exerted the opposite effect on NCI-H520 cells.

### 3.4. LINC01554 Overexpression Suppressed NSCLC Tumour Growth In Vivo

To evaluate the effect of LINC01554 on NSCLC in vivo, we established a xenograft tumour model using nude mice that were injected with H23 cells transfected with pcDNA3.1 LINC01554 or pcDNA3.1 NC. As shown in Figures 3(d)–3(f), LINC01554 overexpression significantly inhibited NSCLC tumour growth (*P* < 0.05).

### 3.5. LINC01554 Expression Directly Targeted miR-1267 Expression, Which in Turn Directly Targeted ING3 Expression

**Figure 4:**
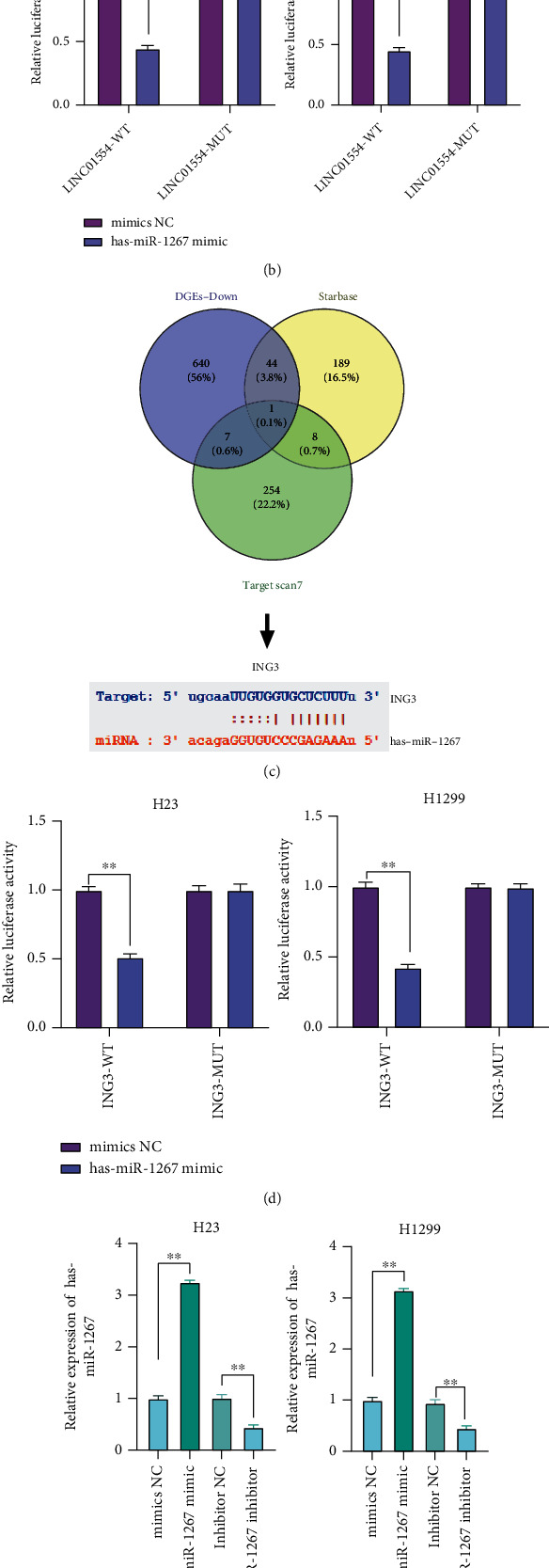
LINC01554 expression directly targeted miR-1267 expression, which in turn directly regulated ING3 expression. (a) Predicted binding sites between LINC01554 and miR-1267. (b) The binding of miR-1267 to LINC01554 was confirmed via the dual-luciferase reporter assay. (c) Predicted binding sites between miR-1267 and ING3. (d) The binding of ING3 to miR-1267 was verified via the dual-luciferase reporter gene assay. (e) Relative expression of miR-1267 in H23 and H1299 cells was examined via RT-qPCR. (f) ING3 expression level was detected via western blotting. ∗∗*P* < 0.01.

To elucidate the potential mechanism by which LINC01554 regulates NSCLC cell proliferation and invasion, we initially predicted the miRNA targets of LINC01554 using the StarBase tool. In addition, we found the expressions of miRNAs that were significantly upregulated from TCGA-LUSC and TCGA-LUAD databases. The significantly upregulated miRNAs overlapped with the predicted miRNA targets of LINC01554, and one potential target miRNA (miR-1267; Figure 4(a)) was identified. Moreover, the dual-luciferase reporter gene assay results showed that the luciferase activity of the wild-type pGL3-promoter LINC01554 3-UTR was significantly decreased in the miR-1267 mimic group, whereas that of the mutant-type pGL3-promoter LINC01554 3-UTR remained unchanged (Figure 4(b), *P* < 0.05). We used TargetScan and StarBase tools to identify the potential downstream target genes of miR-1267. We also collected the genes whose expressions were significantly downregulated from TCGA-LUSC and TCGA-LUAD databases. The significantly downregulated genes overlapped with the predicted downstream target genes of miR-1267, and one potential downstream target gene (*ING3*; Figure 4(c)) was identified. The luciferase activity markedly decreased when the wild-type pGL3-promoter ING3 3-UTR and miR-1267 mimic were cotransfected into cells, as compared with the luciferase activity in negative control cells (Figure 4(d), *P* < 0.05). We overexpressed and inhibited the expression of miR-1267 using the miR-1267 mimic and inhibitor (Figure 4(e)), respectively, and detected the expression of ING3 via western blot analysis. As shown in Figure 4(f), the miR-1267 mimic significantly reduced ING3 expression, and the miR-1267 inhibitor observably elevated ING3 expression (*P* < 0.05).

### 3.6. miR-1267 Overexpression Reversed the LINC01554 Upregulation–Induced Tumour-Suppressive Effects on NSCLC Cells

**Figure 5:**
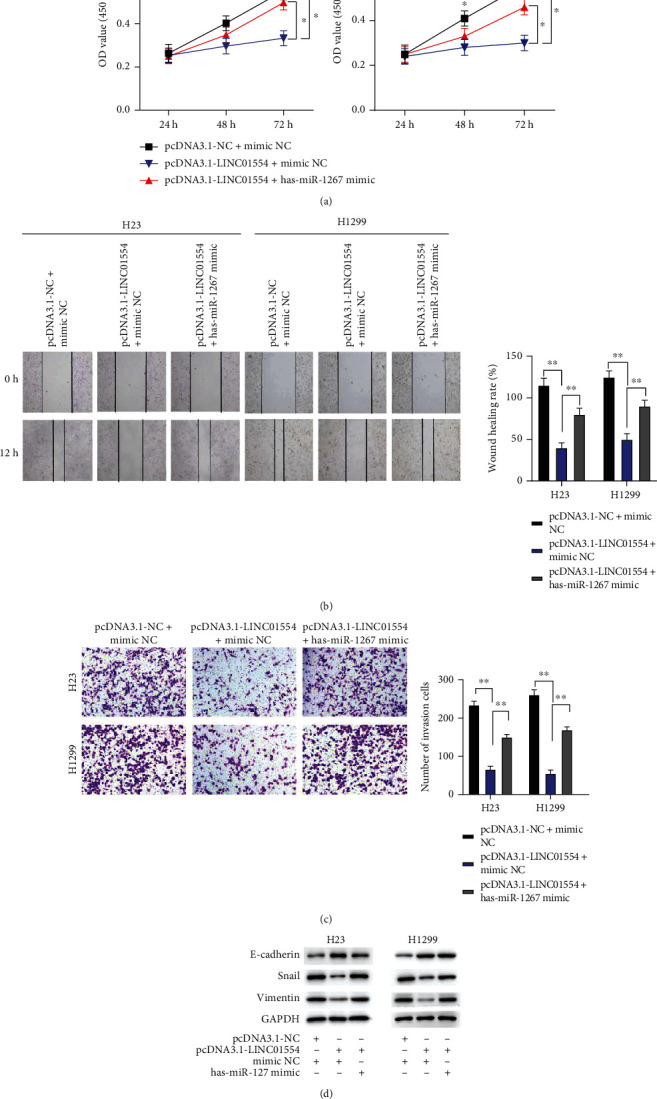
miR-1267 overexpression reversed the LINC01554 upregulation–induced tumour-suppressive effects on NSCLC cells. (a) The vitality of H23 and H1299 cells was tested via the MTT assay. (b) Cell migration was detected and quantitated via the wound healing assay in H23 and H1299 cells. (c) Cell invasion was tested and quantitated via the transwell assay in H23 and H1299 cells. (d) E-cadherin, snail, and vimentin expression were detected via western blotting. ∗*P* < 0.05, and ∗∗*P* < 0.01.

To determine whether LINC01554 contributes to NSCLC progression by targeting miR-1267, we further investigated the function of miR-1267 in NSCLC cells. The MTT assay result indicated that LINC01554 overexpression led to a considerable decrease in the proliferation ability of H23 and H1299 cells, and this effect was abrogated following the cotransfection with pcDNA3.1 LINC01554 and miR-1267 mimic (Figure 5(a), *P* < 0.05). Wound healing and transwell invasion assays revealed that the migration and invasion of NSCLC cells were significantly reduced by LINC01554 overexpression. However, the migration and invasion capabilities of cells were dramatically enhanced following cotransfection with pcDNA3.1 LINC01554 and miR-1267 mimic (Figures 5(b) and 5(c), *P* < 0.05). Likewise, as presented in Figure 5(d), LINC01554 overexpression substantially inhibited the expression of snail and vimentin and improved the expression of E-cadherin in NSCLC cells (*P* < 0.05). In comparison with the NC group, the group cotransfected with pcDNA3.1 LINC01554 and miR-1267 mimic showed significant EMT (Figure 5(d), *P* < 0.05).

### 3.7. Both LINC01554 and miR-1267 Overexpression Modulated the ING3/Akt/mTOR Pathway

**Figure 6:**
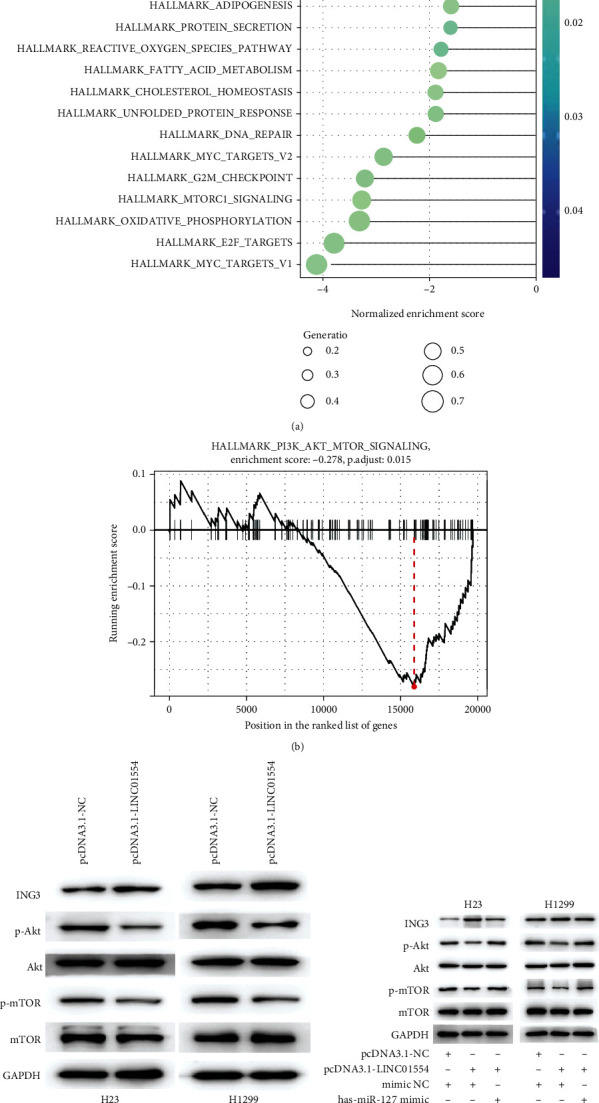
Both LINC01554 and miR-1267 overexpression modulated the ING3/Akt/mTOR pathway. (a, b) LINC01554 was associated with Akt/mTOR signalling. (c) The expression of ING3 and Akt/mTOR pathway-related proteins (Akt, p-Akt, mTOR, and p-mTOR) was detected via western blotting and quantitated in H23 and H1299 cells treated with pcDNA3.1-LINC01554 and the empty pcDNA3.1 vector. (d) The expression of ING3 and Akt/mTOR pathway-related proteins (Akt, p-Akt, mTOR, and p-mTOR) was detected via western blotting and quantitated in H23 and H1299 cells treated with pcDNA3.1-LINC01554 and the empty pcDNA3.1 vector, miR-1267 mimic, and NC mimic.

To understand the underlying mechanism of LINC01554 in NSCLC progression, gene set enrichment analysis (GSEA) was performed on TCGA-LUSD datasets. The results showed that LINC01554 expression was associated with PI3K-Akt-mTOR signalling (Figures 6(a) and 6(b)), thereby indicating that the PI3K-Akt-mTOR pathway might be involved in the growth-inhibiting effect of LINC01554. The overexpression of LINC01554 significantly elevated ING3 expression but suppressed p-Akt and p-mTOR expressions. However, the total level of Akt and mTOR protein remained unchanged (Figure 6(c), *P* < 0.05). Moreover, H23 and H1299 cells were cotransfected with LINC01554 and miR-1267 mimic; as expected, the expression of ING3, p-Akt, and p-mTOR was recovered. The miR-1267 mimic blocked the effect of the upregulated LINC01554 (Figure 6(d), *P* < 0.05).

## 4. Discussion

NSCLC has seriously endangered human health, but due to the complexity of the pathogenesis of NSCLC, there is no effective treatment in clinic [12]. Previous studies have confirmed that dysregulation in lncRNA expression plays a key role in many biological processes in different cancers, including NSCLC. In our study, results showed that NSCLC cell proliferation could be inhibited by overexpressed LINC01554 both *in vivo* and *in vitro*. The above results were in accordance with the previous study [2, 12, 19, 20], which indicated that LINC01554 played a tumour suppressive role in NSCLC development.

Recently, the research on lncRNA-miRNA-mRNA ceRNA network provides a basis for a better understanding of the role of lncRNA-miRNA interaction in mRNA regulation and NSCLC development [21, 22]. miRNAs can interact with target mRNA to degrade mRNA or inhibit mRNA translation [7]. Compared to miRNAs, lncRNAs are longer ncRNAs that play a role through more diverse mechanisms [23]. In addition to directly targeting mRNAs, it has also been proved to have the function of ceRNAs, which interact with miRNAs and indirectly regulate mRNAs [24]. Based on bioinformatic analysis and dual-luciferase assay results, we confirmed that LINC01554 directly targets miR-1267 and that miR-1267 in turn regulates ING3 expression. miR-1267 was reported to be upregulated in breast cancer, and its expression was significantly related to cancer grade and stage [25]. A recent study stated that the lncRNA SNHG11 is involved in the progression of gastric cancer through regulation of the expression of catenin-*β*1 and autophagy-related 12 via miR-1276 [26]. However, the biological function of miR-1267 in NSCLC progression has not been clarified. Notably, in this study, we found that miR-1267 overexpression reversed the LINC01554 upregulation–induced tumour-suppressive effects on NSCLC cells and in line with the previous study.

As a crucial inhibitor of the growth gene family, ING3 is usually abnormally expressed in both the cytoplasm and nucleus of tumour cells and has been shown to be involved in the progression of many tumours. Studies have shown that ING3 regulates cell proliferation, apoptosis, and cell cycle arrest in gastric cancer by inactivating the PI3K/Akt pathway and promoting cell death [27]. Downregulation of ING3 expression and its transfer to the cytoplasm can regulate cell cycle arrest, senescence, and apoptosis in head and neck squamous cell carcinoma [28]. ING3 level is markedly decreased in breast cancer where its expression is closely correlated with cancer prognosis [29]. These results are consistent with our results, which indicate that LINC01554 might directly regulate ING3 through miR-1267 and modulate the proliferation, migration, invasion, and EMT of NSCLC cells.

Abnormal expression of lncRNAs has been also reported to be involved in the progression of NSCLC through inactivation or activation of the PI3K/Akt signalling pathway. For instance, the lncRNA HULC facilitates NSCLC cell growth and suppresses cell apoptosis by stimulating the PI3K/Akt signalling pathway [30]. HOXB-AS3 stimulates NSCLC cell proliferation, migration, and invasion by dysregulating the PI3K/Akt signalling pathway [31]. The lncRNA TBX5-AS1 modulates tumour progression in NSCLC by suppressing the PI3K/Akt signalling pathway [32]. To identify the downstream signal pathway of LINC01554 in anti-NSCLC, we performed a GSEA analysis. The results showed that LINC01554 could inhibit Akt/mTOR pathway. Collectively, overregulation of LINC01554 inhibits cell viability, migration, and proliferation by inhibiting the Akt/mTOR pathway.

In summary, we report for the first time that LINC01554 directly regulates ING3 expression through miR-1267 and thus affects the proliferation, migration, invasion, and EMT of NSCLC cells by regulating the Akt/mTOR pathway. LINC01554 may function as a novel prospective therapeutic target for the treatment of NSCLC.

## Data Availability

The data that support the findings of this study are available from the corresponding author upon reasonable request.
